# Causal association between adiponectin and the risk of Alzheimer's disease: A Mendelian randomization study

**DOI:** 10.3389/fneur.2022.1038975

**Published:** 2022-12-09

**Authors:** Tianyu Jin, Wei Huang, Fangzheng Cao, Xinyue Yu, Zhenhua Ying, Shunyuan Guo, Yifan Cheng, Chao Xu

**Affiliations:** ^1^The Second Clinical Medical College, Zhejiang Chinese Medical University, Hangzhou, China; ^2^Center for Rehabilitation Medicine, Department of Neurology, Zhejiang Provincial People's Hospital, Affiliated People's Hospital, Hangzhou Medical College, Hangzhou, China; ^3^Rheumatism and Immunity Research Institute, Zhejiang Provincial People's Hospital, Affiliated People's Hospital, Hangzhou Medical College, Hangzhou, China; ^4^Alberta Institute, Wenzhou Medical University, Wenzhou, China

**Keywords:** Alzheimer's disease, adiponectin, causality, Mendelian randomization, risk

## Abstract

**Background:**

Numerous observational studies have revealed that circulating adiponectin (ADPN) is associated with Alzheimer's disease (AD) risk. However, the causality remains unknown. We aimed to assess the causality of circulating ADPN on AD risk using Mendelian randomization (MR).

**Methods:**

Fourteen single nucleotide polymorphisms (SNPs) significantly associated with ADPN were selected from publicly available genetic abstract data. We applied these SNPs to two recent large-scale genome-wide association studies (GWAS) of AD, one from the FinnGen consortium and the other from a large meta-analysis. The inverse variance weighted method, MR–Egger method, the weighted median method, the Cochran Q statistic, the MR-Pleiotropy Residual Sum and Outlier methods, and the leave-one-out analysis were applied for MR analyses.

**Results:**

In MR analysis, no significant genetic association was found between plasma ADPN levels and AD risk by analyzing the FinnGen consortium GWAS database in the inverse variance weighted method [odds ratio (OR): 0.874, 95% confidence interval (CI): 0.701–1.089, *p* = 0.230], MR–Egger (OR: 0.944, 95% CI: 0.692–1.288, *p* = 0.721), and weighted median method (OR: 0.900, 95% CI: 0.678–1.194, *p* = 0.449). Additionally, the same analysis was conducted for the meta-analysis database, and we found no significant association (OR: 1.000, 95% CI: 0.999–1.001, *p* = 0.683).

**Conclusion:**

Our findings reveal no significant causal association between circulating ADPN and AD risk.

## Introduction

Alzheimer's disease (AD), which accounts for ~50–70% of the types of dementia, is defined as a person having a significant decline in cognition and behavior resulting in interference with family, occupational, or social functioning (1). The prevalence of AD is estimated to be as high as 5–7% of individuals over 65 globally and is increasing yearly (2). The growing prevalence of AD is undoubtedly placing enormous pressure on families, health-care professionals, society, and governments. Several risk factors, such as advanced age, sex, family history, obesity, chronic inflammation, diabetes, and hyperlipidemia, are important risk factors for the development and progression of AD (3, 4). However, a portion of the risk factors remains unclear.

Numerous epidemiological and experimental studies have shown that patients with metabolic risk factors such as dyslipidemia, type 2 diabetes mellitus (T2DM), and homocysteinemia are more likely to develop AD, while much less is known about the role of adipokines, such as adiponectin (ADPN), in this regard (5–7). ADPN is a monomeric glycoprotein secreted by adipocytes (8). It has a variety of biological activities, including improving insulin resistance, reducing inflammation, and preventing atherosclerosis (9). Therefore, ADPN is a protective factor against T2DM, obesity, chronic inflammation, and cardiovascular diseases (10). Several observational studies have been conducted on ADPN and the risk of AD (11–14). However, the conclusions are not consistent. According to a study by Teixeira et al. (15), AD patients' circulating ADPN levels were significantly lower than those in healthy elderly people. Nevertheless, some studies have not found a significant association between healthy controls and ADPN levels (11–14). Previous epidemiological studies had some limitations, such as the possibility of reverse causality and interference from potential confounding factors. Thus, it is still unclear whether plasma levels of ADPN have an effect on the risk of AD.

Indeed, previous observational studies have shown strong associations between various risk factors and disease, while subsequent findings have revealed that these factors are not causal but rather due to the interference of residual confounding factors. Some prominent examples include the associations between beta carotene and vitamin A and lung cancer (16, 17), vitamin E consumption and coronary heart disease (18, 19), and estrogen plus progestin and cardiovascular disease (20, 21). The Mendelian randomization (MR) method is an essential statistical method that utilizes instrumental variables (IVs) to study the genetic causality of exposure and outcome (22–25). Some examples include causal associations between alcohol consumption and AD (26), herpesvirus infections and AD (27), and tea intake and Parkinson's disease (28). Compared with traditional observational studies, MR analysis is able to overcome reverse causation, as genes are relatively stable after fertilization (29). In addition, it can eliminate residual confounding interference due to the random assignment of alleles (30).

In this study, we applied a two-sample MR analysis using data based on large-scale genome-wide association studies (GWAS) to explore the causal association between circulating ADPN and the risk of AD.

## Methods

### Study design

**Figure 1:**
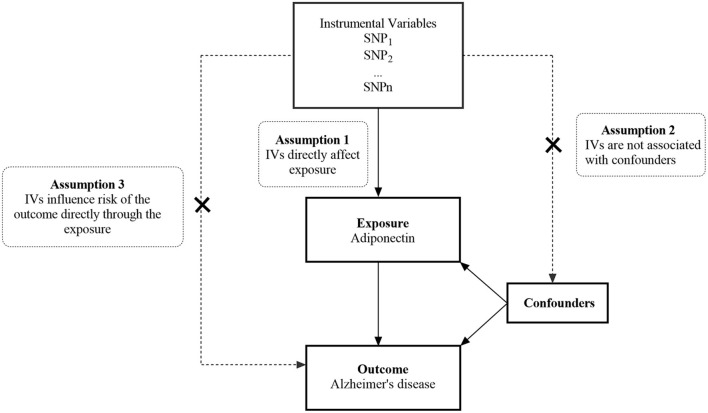
An overview of the study design. SNP, single nucleotide polymorphism; IVs, instrumental variables.

We performed a two-sample MR study using the publicly available GWAS catalog. Ethical approval and consent were provided in the original publication. An overview of this research design is shown in Figure 1. The MR study has the following three core assumptions (31, 32): (1) The selected IVs should be significantly associated with exposure (ADPN); (2) The selected IVs are not related to confounding factors of outcome (AD); and (3) The selected IVs affect the outcome through exposure directly rather than through other pathways.

### GWAS data for adiponectin

We searched the GWAS catalog to obtain single nucleotide polymorphisms (SNPs) as IVs. The SNPs in our study had genome-wide significance (*p* < 5 × 10^−8^) and a threshold of linkage disequilibrium (LD) *r*^2^ < 0.001 within a 10,000-kilobase window.

Our primary IVs were obtained from the pooled data of the latest GWAS meta-analysis on circulating ADPN from the ADIPOGen consortium (33). A total of 39,883 participants of European ancestry were included in this meta-analysis. PhenoScannerV2 (www.phenoscanner.medschl.cam.ac.uk) was employed to further assess the potential association of IVs with confounders of AD risk (34). We finally included 14 SNPs significantly associated with circulating ADPN (*p* < 5 × 10^−8^, LD *r*^2^ < 0.001). Eventually, the strength of the included SNPs was estimated using F-statistics (35).

### GWAS data for AD

The GWAS data for AD were extracted from the FinnGen consortium and a large meta-analysis by Kunkle et al. (36). In the FinnGen database, we used the R5 version of the AD database, in which there were 115,370 participants of European ancestry, including 3,899 cases and 111,471 controls. The International Classification of Diseases codes defined diagnostic criteria for AD. To verify the accuracy of the results, we additionally included the meta-analysis by Kunkle et al. (36), which was comprised of 21,982 cases and 41,944 controls from the European population (36). Participants were certified by a specialist in neurology. The GWAS data source details in our study are shown in Table 1.

**Table 1:** Details of the GWAS included in the Mendelian randomization.

**Consortium**	**Year**	**Trait**	**Sample size**	**Web source**
A meta-analysis of GWAS	2012	Adiponectin	39,883	doi: 10.1371/journal.pgen.1002607
A meta-analysis of GWAS	2022	Alzheimer's disease	488,285	doi: 10.3390/nu14091697
FinnGen	2021	Alzheimer's disease	115,370	https://www.finngen.fi/en

GWAS, Genome-Wide Association Studies.

### Statistical analysis

Our MR analysis was implemented with the TwoSampleMR (version 0.5.5) and MRPRESSO (version 1.0) packages in R (version 4.2.1).

We harmonized the data of the exposure and outcome to keep the effect allele associated with the same allele. The inverse variance weighted (IVW) method, the weighted median method (WM), and the MR–Egger method were used to evaluate the MR estimates of circulating ADPN for the risk of AD (37). The IVW method was the main result because it was the Wald ratio of individual SNPs for meta-analysis, which assumes that all the included IVs were valid (38). In addition, WM and MR–Egger approaches were applied to complement IVW estimation. Although these approaches have broader confidence intervals (CIs), they can provide more reliable estimates in a wider range of scenarios (39). If the results of these methods were inconsistent, we tightened the IVs and reanalyzed them.

Sensitivity analyses were performed to examine horizontal pleiotropy and heterogeneity in violation of the MR assumptions. We used Cochran's *Q*-test to evaluate the heterogeneity of effect sizes generated by the selected genetic IVs (40). If Cochran's *Q*-test shows a *p* < 0.05, it indicates the presence of potential heterogeneity. The MR-Pleiotropy Residual Sum and Outlier (MR-PRESSO) method was applied to detect and adjust for potential horizontal polymorphism (24, 41). The intercept of MR–Egger regression provides an assessment of horizontal polymorphism (*p* < 0.05 indicates the presence of horizontal polymorphism) (39). The leave-one-out analysis was used to investigate the overall impact of individual SNPs (42).

We believe that a relatively robust causal association requires satisfying the following items:

1. The IVW, WM, and MR–Egger methods presented directionally consistent causal estimates.
2. The intercept derived from the MR–Egger regression did not reveal directionally detected polymorphisms (*p* > 0.05).
3. Cochran's *Q*-test indicated no significant heterogeneity (*p* > 0.05).
4. The leave-one-out analysis showed that each SNP did not significantly influence the causal estimate.

## Results

### Genetic instrumental variant selection

Overall, 14 SNPs were used for the causal association analysis of circulating ADPN with the risk of AD. The F-statistics for these SNPs ranged from 10.06 to 98.72, indicating that the weak instrumental bias was unsupported. Specific SNP information, including effect allele, other alleles, effect allele frequency, beta (β), standard error, *P*-value, and *R*^2^ and F-statistic, are detailed in Table 2.

**Table 2:** Characteristics of instrumental variables for circulating adiponectin.

**SNP**	**Trait**	**EA**	**OA**	**Samplesize**	**β**	**EAF**	**SE**	***P*-value**	***R* ^2^**	**F statistic**
rs2062632	Adiponectin	C	T	29,028	−0.0546	0.6864	0.0058	2.52E-19	0.00129	37.39
rs17366568	Adiponectin	A	G	24,865	−0.1541	0.9083	0.0087	1.00E-200	0.00395	98.72
rs1108842	Adiponectin	C	A	29,338	0.0299	0.4583	0.0044	3.66E-11	0.00044	13.05
rs1597466	Adiponectin	T	G	29,319	−0.0477	0.0920	0.0075	1.89E-08	0.00037	11.13
rs6810075	Adiponectin	C	T	29,140	−0.0664	0.6333	0.0048	1.00E-200	0.00205	59.79
rs7615090	Adiponectin	G	T	21,869	−0.0581	0.8833	0.0085	2.81E-11	0.00070	15.25
rs2980879	Adiponectin, triglycerides, high-density lipoprotein	T	A	24,084	0.0299	0.3750	0.0051	1.08E-08	0.00041	10.06
rs7955516	Adiponectin	C	A	29,178	0.0264	0.4417	0.0046	2.43E-08	0.00034	10.07
rs601339	Adiponectin	G	A	29,325	0.0390	0.1500	0.0057	3.87E-11	0.00038	11.38
rs7964945	Adiponectin	A	T	29,252	0.0368	0.8083	0.0064	2.61E-08	0.00042	12.33
rs8042532	Adiponectin	G	T	7,850	−0.3397	0.9917	0.0554	2.86E-09	0.00191	15.00
rs12051272	Adiponectin	T	G	15,593	−0.2765	0.0090	0.0181	1.00E-200	0.00138	21.51
rs2927324	Adiponectin	T	C	29,184	0.0315	0.4746	0.0045	1.29E-11	0.00050	14.46
rs731839	Adiponectin	A	G	29,166	0.0366	0.6724	0.0048	2.20E-13	0.00059	17.24

EA, effect allele; OA, other allele; EAF, effect allele frequency; SE, standard error.

### MR estimates and sensitivity analyses of the FinnGen consortium database

**Figure 2:**
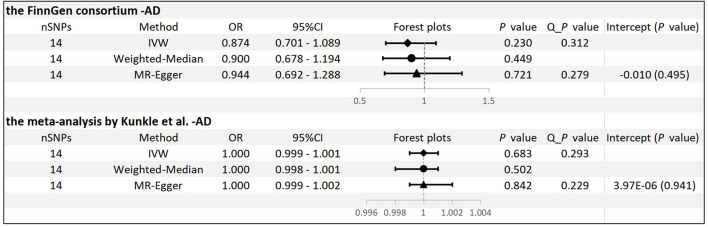
MR results and sensitivity analysis for association of ADPN and AD. IVW, inverse variance weighted.

No significant differences were found between circulating ADPN and AD risk by analyzing the FinnGen consortium GWAS database in the IVW method [odds ratio (OR): 0.874, 95% CI: 0.701–1.089, *p* = 0.230]. Consistent conclusions were also obtained using the MR–Egger method (OR: 0.944, 95% CI: 0.692–1.288, *p* = 0.721) and the WM method (OR: 0.900, 95% CI: 0.678–1.194, *p* = 0.449) (Supplementary Figure 1). Moreover, Cochran's *Q*-test showed no evidence of significant heterogeneity in our study (*p* = 0.312 for IVW and *p* = 0.279 for MR–Egger). MR-PRESSO also presented similar results (global test *p* = 0.370) (Figure 2). The leave-one-out analysis found no individual SNP that significantly affected the risk of AD by circulating ADPN, which indicates that the results were reliable (*p* = 0.230) (Supplementary Figure 2).

### MR estimates and sensitivity analyses of the meta-analysis

We conducted the same analysis for the database from the meta-analysis by Kunkle et al. and did not find a significant association between ADPN and AD (OR: 1.000, 95% CI: 0.999–1.001, *p* = 0.683). Similar risk estimates were obtained employing MR–Egger (OR: 1.000, 95% CI: 0.999–1.002, *p* = 0.842) and WM (OR: 1.000, 95% CI: 0.998-1.001, *p* = 0.502) (Supplementary Figure 3). In addition, there was no evidence of significant horizontal pleiotropy or significant heterogeneity (Figure 2). The leave-one-out analysis also found no individual SNP that significantly drives the estimates of the risk of AD on circulating ADPN (*p* = 0.683) (Supplementary Figure 4).

## Discussion

In this study, we explored the causality between circulating ADPN and AD risk using the MR study based on two GWAS databases of AD and one database of ADPN. According to the latest available public databases, our findings did not support a significant genetic relationship between circulating ADPN and AD risk.

Until now, current studies on the association between circulating ADPN and AD risk have been limited to observational studies, and the causality has not been clarified. A recent case–control study by Gorska-Ciebiada et al. (12) found significantly lower levels of circulating ADPN in patients wth AD and mild cognitive impairment (MCI) than in those without dementia. However, a prospective cohort study that included 541 women with a mean follow-up of 13 years revealed that high levels of plasma ADPN levels were an independent risk factor for AD (HR = 1.87, 95% CI: 1.13–3.10) (43). Some observational studies have shown no significant association between ADPN and AD risk (13, 44, 45). The divergent conclusions in these studies might be due to inconsistent diagnostic criteria for AD and failure to exclude confounding factors that have an impact on ADPN. In addition, most previous observational studies were unable to reveal a causal association between circulating ADPN and AD risk.

We performed a functional examination of the included SNPs on PhenoScannerV2 and found that SNP rs2980879 was associated with ADPN, triglycerides, and high-density lipoprotein (34). Therefore, some sensitivity analyses were performed to ensure the reliability of our results. MR-PRESSO, MR–Egger regression, and the leave-one-out analysis proved that our results were stable and reliable.

Although our study showed no causal relationship between ADPN and AD risk, the serum levels of ADPN may be associated with the severity of AD. A case–control study by Khemka et al. found that serum ADPN levels showed a significant negative association with MMSE scores in 196 patients with AD (*p* < 0.001) (14). In fact, it is widely accepted that there is a potential role of plasma ADPN levels in AD. First, ADPN improves prominence regulation, which promotes the growth of neural prominences and increases synaptic plasticity, further enhancing hippocampal function (46). Moreover, an animal study showed that deficiency in ADPN in mice leads to cognitive impairment and AD-like pathology (47). Second, ADPN is one of the classic anti-inflammatory agents (9). It is well-known that the critical factor of dementia is a long-term chronic inflammation of the central nervous system (CNS) (48). The leading cause of CNS chronic inflammation is the release of inflammatory factors by microglia, such as interleukin-1 (IL-1), interleukin-6 (IL-6), and tumor necrosis factor-α (TNF-α) (49, 50). ADPN can reduce the phenotype of the proinflammatory effects of microglia and macrophages through the AdipoR1/NF-κB signaling pathway (51, 52). Therefore, increased levels of ADPN might reduce neuroinflammation in AD. Third, a number of studies have shown that T2DM is an independent risk factor for dementia (6, 53, 54). In addition, insulin signaling pathways also play a crucial role in MCI and dementia (55, 56). ADPN is a potential therapeutic target for T2DM, and the main mechanism is to regulate blood glucose by improving insulin resistance. It is currently used for the clinical treatment of patients with T2DM (10, 57, 58).

Our research has several strengths. First, MR studies can mimic RCTs, which are widely considered a way to reveal causality and avoid reverse causality. At the same time, compared to RCT studies, MR studies are not as costly and labor-intensive. Second, MR studies can effectively prevent the influence of confounding factors due to the random assignment of alleles. Third, we strictly screened for relevant SNPs using Plink clumping and PhenoscannerV2, which are not weak instruments in F statistics.

However, some limitations exist in our MR study. First, since the GWAS databases in our study were all from European populations, whether this causal association remains insignificant in other populations needs further investigation. Second, we could not obtain detailed demographic and clinical data on the participants. Therefore, we could not perform further subgroup analysis. Third, our MR study evaluated the effects of lifetime exposure, which might be overestimated in the real world if effective interventions exist. Finally, epigenetic issues such as genomic imprinting, maternal effects, and gene silencing are unavoidable weaknesses of MR analysis and may introduce bias.

## Conclusion

In summary, this is the first MR study to explore the genetic association between ADPN and the risk of AD. The results of our MR study do not support the hypothesis that circulating ADPN may reduce the risk of AD, and further studies are needed to verify our results in the real world.

## Data availability statement

The original contributions presented in the study are included in the article/Supplementary material, further inquiries can be directed to the corresponding author/s.

## Ethics statement

Ethical approval and consent had been provided in the original publication. The patients/participants provided their written informed consent to participate in this study.

## Author contributions

TJ presented the ideas, performed the MR analysis, evaluateed the quality of the study, and drafted the manuscript. WH evaluated the quality of the study and revised the manuscript. FC and ZY wrote the statistical analysis plan and conducted the quality assessment. XY drew figures and conducted the quality assessment and plot tables. SG revised the manuscript and assisted with funding. YC and CX carried out the supervision, checked and verified all data, and final approvement. All authors contributed to the article and approved the submitted version.
