# Supplementary material for: Causal association between adiponectin and the risk of Alzheimer's disease: A Mendelian randomization study
Source: Front Neurol. 2022 Dec 9;13:1038975. doi: 10.3389/fneur.2022.1038975 (PMC9780387; doi:10.3389/fneur.2022.1038975)
Supplement: Supplementary file 1 [file Data_Sheet_1.docx]

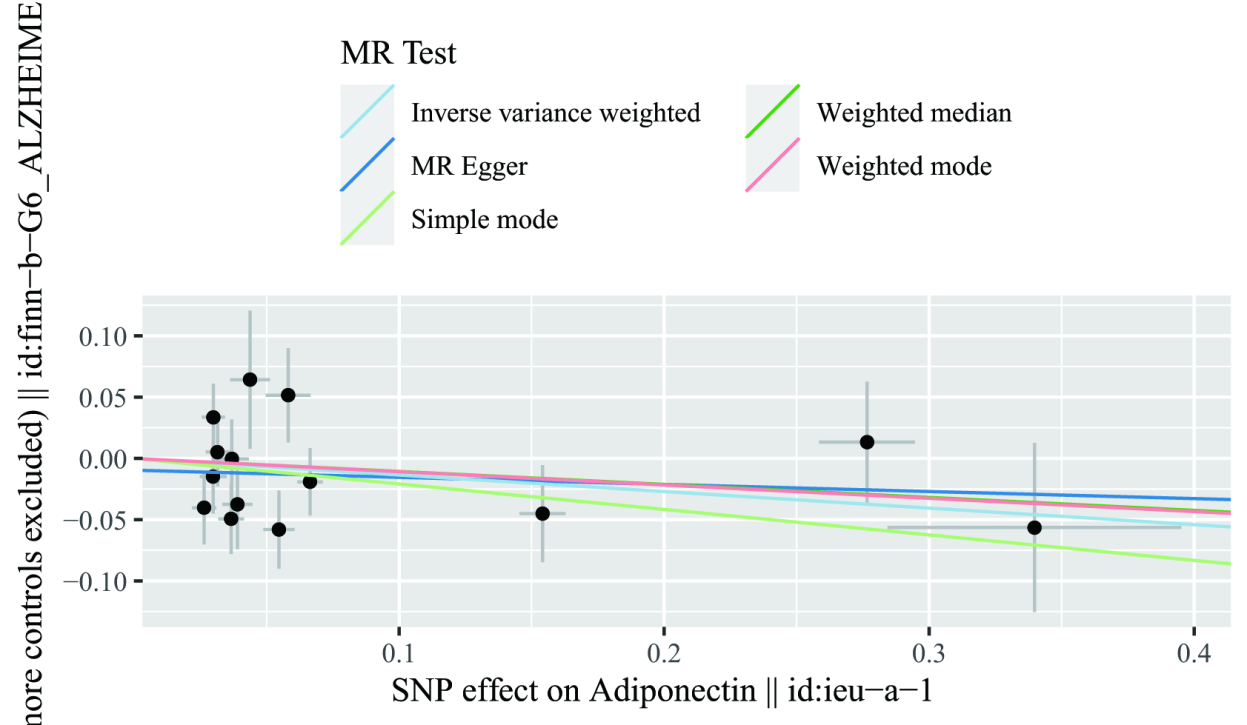


Supplementary Figure 1. FinnGen database: MR results for association of ADPN and AD. The lines indicate the estimated effect sizes by five Mendelian randomization methods (inverse‐variance weighted (IVW), MR Egger, Simple mode, Weighted mode and weighted median). The IVW method (OR=0.874, 95%CI:0.701-1.089, *p*=0.230), MR-Egger method (OR=0.944, 95%CI: 0.692-1.288, *p*=0.721), and WM method (OR=0.900, 95%CI:0.678-1.194, *p*=0.449) found no significant association between circulating ADPN and AD risk.


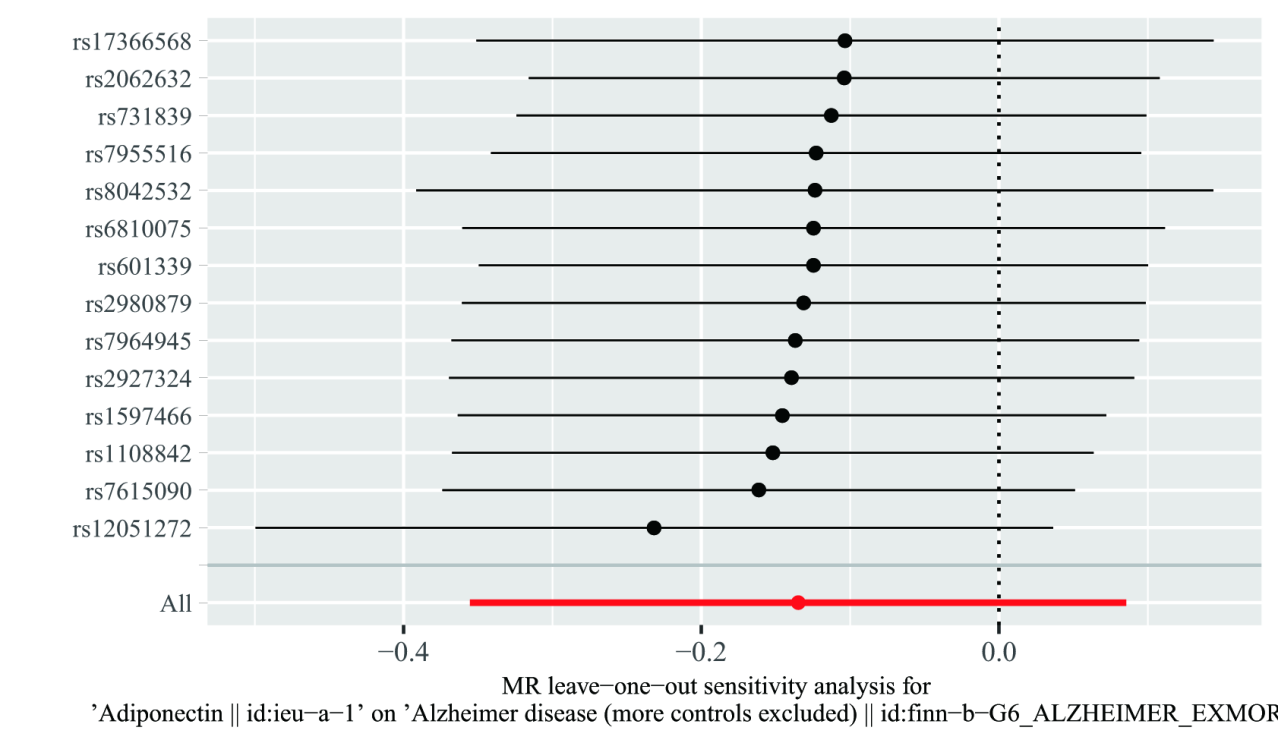


Supplementary Figure 2. FinnGen database: MR leave-one-out sensitivity analysis of ADPN and AD. The leave-one-out plot visualized how the causal estimates (point with horizontal line) for the effect of ADPN on AD were influenced by the removal of single variant. The leave-one-out analysis found no individual SNP that significantly affected the risk of AD by circulating ADPN, which indicates that the results were reliable (p=0.230).


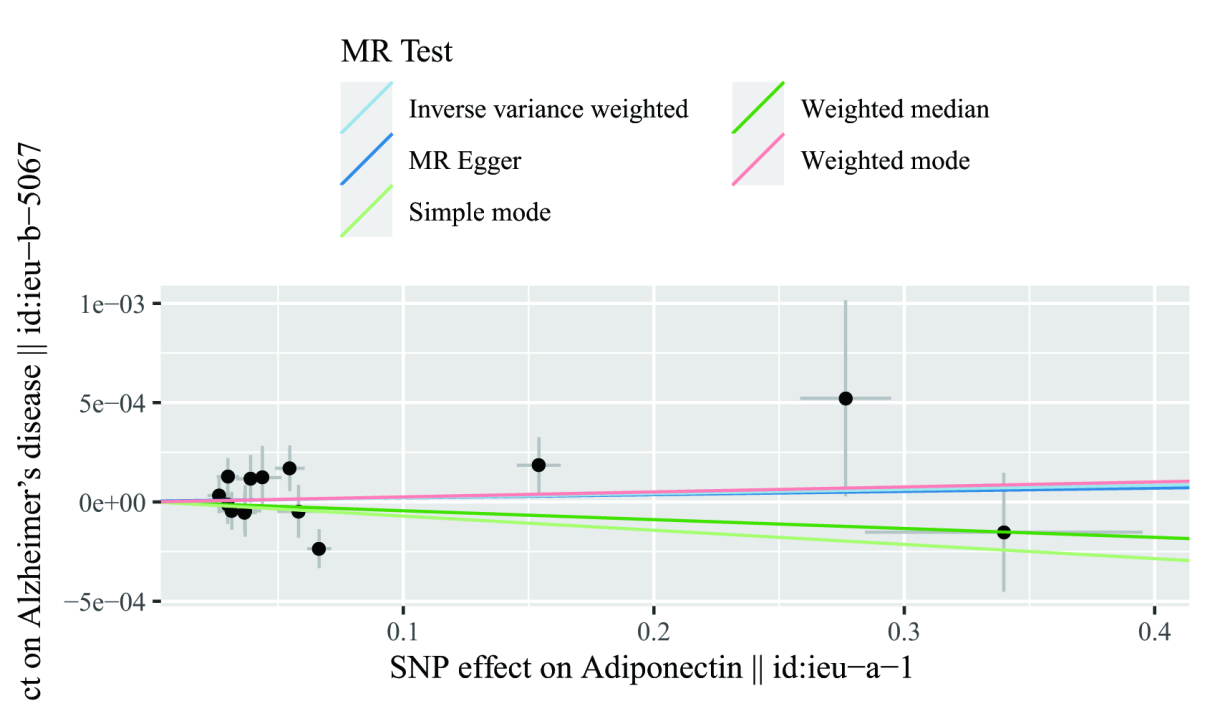


Supplementary Figure 3. Meta-analysis by Kunkle et al. database: MR results for association of ADPN and AD. The IVW method (OR=1.000, 95%CI:0.999-1.001, p=0.683), MR-Egger (OR=1.000, 95%CI:0.999-1.002, p=0.842) and WM (OR=1.000, 95%CI:0.998-1.001, p=0.502) found no significant association between circulating ADPN and AD risk.


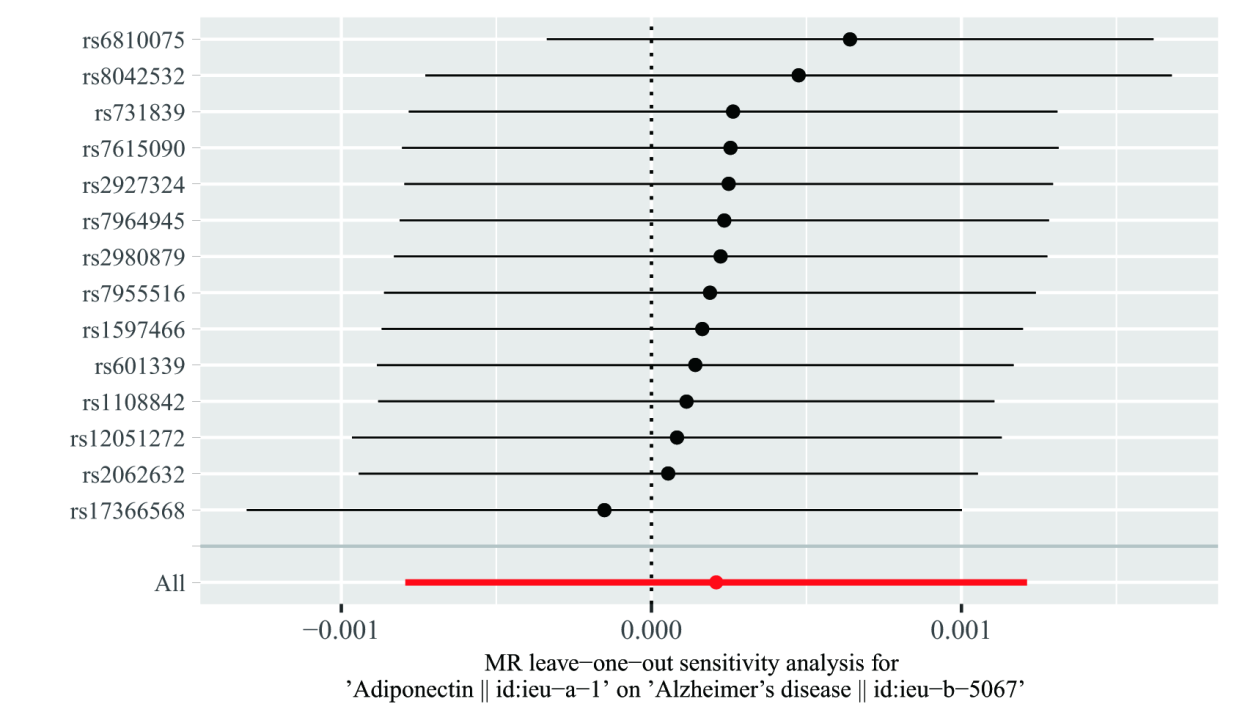


Supplementary Figure 4. Meta-analysis by Kunkle et al. database: MR leave-one-out sensitivity analysis of ADPN and AD. The leave-one-out plot visualized how the causal estimates (point with horizontal line) for the effect of ADPN on AD were influenced by the removal of single variant. The leave-one-out analysis found no individual SNP that significantly affected the risk of AD by circulating ADPN, which indicates that the results were reliable (p=0.683).
